# Global Trends and Developments in Diet and Longevity Research: A Bibliometric Analysis

**DOI:** 10.3390/nu17132119

**Published:** 2025-06-26

**Authors:** Simge Sipahi, Kezban Esen Karaca Çelik, Nurhan Doğan, Theodora Mouratidou, Murat Baş

**Affiliations:** 1 Department of Nutrition and Dietetics, Faculty of Health Sciences, Acibadem Mehmet Ali Aydinlar University, 34752 Istanbul, Turkey; murat.bas@acibadem.edu.tr; 2 Department of Nutrition and Dietetics, Faculty of Health Sciences, Izmir Demokrasi University, 35140 Izmir, Turkey; esen.karaca@idu.edu.tr; 3 Department of Biostatistics and Medical Informatics, Faculty of Medicine, Afyonkarahisar Health Sciences University, 03030 Afyonkarahisar, Turkey; nurhandogan@hotmail.com; 4 Department of Nutrition and Dietetic Sciences, School of Health Sciences, Hellenic Mediterranean University, 72300 Sitia, Greece; tmouratidou@hmu.gr

**Keywords:** diet, longevity, healthy aging, bibliometric analysis, VOSviewer, InCites

## Abstract

**Background/Objectives**: The global population is rapidly aging, raising interest in dietary practices for promoting the healthspan. This study aimed to comprehensively investigate the state of diet and longevity research over the past decade, addressing the lack of bibliometric synthesis within the field. **Methods**: A bibliometric analysis was performed using the keywords “diet” and “longevity” on English-language articles from the Web of Science database that were published from 2015 to 2024. Data were analyzed using Web of Science tools, InCites, and VOSviewer to identify trends in publication output, citation metrics, coauthorship networks, institutional contributions, and keyword co-occurrence patterns. **Results**: Overall, 2203 articles meeting the inclusion criteria were included in the analysis. Publication volume and citation counts gradually increased, peaking in 2021. Countries, including the United Kingdom, and organizations, such as the National Institutes of Health and Harvard University, had significant citation impact, and the United States and China led productivity. Molecular processes (oxidative stress and autophagy), dietary models (Mediterranean diet and calorie restriction), and public health issues (obesity and mortality) were the main thematic clusters. Model species, including *Caenorhabditis elegans* and *Drosophila melanogaster*, were frequently used. Regional disparities in research production and notable terminology variability were noted. **Conclusions**: This study emphasizes the development and diversity of nutrition and longevity research while highlighting novel molecular and translational topics. More international cooperation, uniform language, and multidisciplinary frameworks are warranted to promote equal scientific advancement worldwide and connect mechanistic discoveries with therapeutic outcomes.

## 1. Introduction

Aging is an inevitable process that can be described as a general and progressive decline in the functions of an organism leading to a reduced ability to maintain homeostasis and respond adaptively to changes. The disruption of physiological systems and the loss of the dynamic balance of homeostasis [1,2] contribute significantly to the development of chronic diseases [3,4]. Lopez-Otín et al. initially proposed 9 hallmarks of aging; however, the concept has since evolved, and currently, 12 hallmarks are recognized, namely, intracellular communication, stem cell depletion, cellular senescence, mitochondrial dysfunction, genomic instability, dysbiosis, chronic inflammation, deregulated nutrient-sensing, disabled macroautophagy, loss of proteostasis, epigenetic alterations, and telomere attrition [5,6]. Successful aging is achieved by possessing and sustaining good health, with adequate homeostasis in alignment with the proper responses of the organism while experiencing the ongoing changes [1,7]. Among the multifactorial causes of the disruption of homeostatic balance and its subsequent progressive deterioration, diet plays a crucial role [4]. Despite a wealth of scientific evidence, the type, quantity, and mix of nutrients that promote long life remain debated [8].

According to the United Nations (UN) World Population Prospects 2024, the number of individuals aged ≥65 years is anticipated to double from 761 million in 2021 to 1.6 billion in 2050, with the proportion of this age group increasing from 10% to 16% globally over that period [9]. The aging of the population is a significant demographic shift that is accompanied by possibilities and difficulties. This demographic shift can be a positive development for healthcare and socioeconomic advancement [10]. Meanwhile, the aging population represents a group of individuals with a higher prevalence of chronic diseases (e.g., diabetes, cardiovascular diseases, cancer, dementia, and other age-related conditions). This expanding aging population provides possibilities for improvements in healthcare and socioeconomic development as well as underscores the urgent need to move the emphasis from merely extending the lifespan to promoting concepts, including “healthy aging” and “healthspan”, which are fundamental pillars of the longevity framework. The healthspan can be defined as the length of time an individual maintains good health and functional capacity [8], whereas the National Academy of Medicine defines healthy longevity as “the state in which years in good health (a state of complete physical, mental, and social well-being) approach the biological lifespan, with physical, cognitive, and social functioning enabling well-being across populations” [11]. There has been growing interest related to the concept of longevity and research aimed at enhancing it. Altering the extent, type, and timing of food consumption is considered one of the most efficient, useful, and safe ways to enhance health, extend the lifespan, and improve the healthspan across species, from bacteria to humans [8]. To date, no bibliometric study has provided a comprehensive overview of the scientific landscape surrounding diet and longevity research.

Here, a comprehensive bibliometric analysis was conducted to explore the body of scientific literature on diet and longevity from 2015 to 2024. The primary aim was to assess and determine publication trends, influential authors, leading institutions, and research areas within this field. The findings of this study may be valuable for researchers, policymakers, and funding agencies in comprehending current directions, exploring research gaps, and directing future studies in the field.

## 2. Materials and Methods

### 2.1. Sourcing and Search Strategy

**Figure 1:**
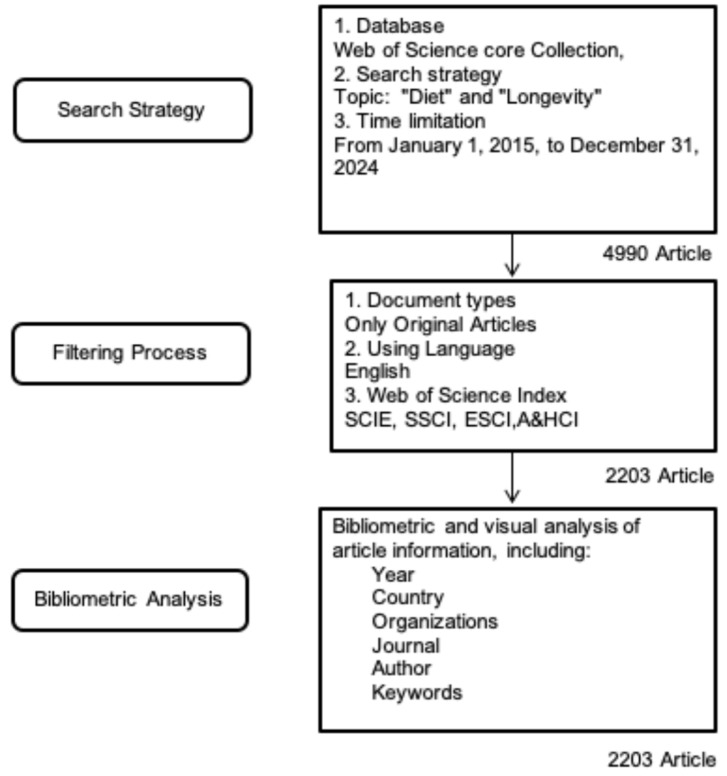
Workflow of the bibliometric study: search strategy, filtering criteria, and analytical framework.

For data collection, a search was conducted on the Web of Science database using the keywords “diet” and “longevity”. The search strategy only used the specific terms “diet” and “longevity”; therefore, no keyword normalization procedures (such as synonym merging or spelling unification) were used. The search was restricted to English-language publications, limited to articles, and covered the period from 2015 to 2024. Data retrieval and export were completed within a single day (25 March 2025) to minimize potential biases due to database updates. The study selection process is visually represented in the flow diagram (Figure 1).

### 2.2. Inclusion Criteria

The inclusion criteria were as follows: studies published in English from 2015 to 2024 containing keywords including “diet” and “longevity” (searching title, abstract, and author keywords). The following exclusion criteria were applied: (1) publications not in English, (2) document types other than original research articles (e.g., reviews, editorials, conference abstracts), and (3) studies published outside the target timeframe (2015–2024).

### 2.3. Data Retrieval and Analysis

The study began with preliminary bibliometric analyses, including publication counts, citation metrics, and global research trends, using the built-in tools available on the Web of Science platform. To facilitate more sophisticated analyses, such as mapping academic collaborations and identifying key research themes, the dataset was exported to InCites, an advanced tool for research performance evaluation. Category Normalized Citation Impact (CNCI) and Journal Normalized Citation Impact (JNCI) metrics were obtained through Clarivate’s InCites platform, which automatically computes these indicators based on the Web of Science Core Collection. The CNCI measures citation performance by comparing the actual number of citations an article receives to the expected number of citations for documents of the same type (article, review), year, and subject category. Similarly, the JNCI normalizes citation impact at the journal level by comparing an article’s citation count to the average citation performance of all documents in the same journal and year. In both metrics, a value of 1.0 indicates world-average performance, while values greater than 1.0 suggest above-average impact. For example, a CNCI of 2.0 means the article is cited twice as much as expected based on its field and year. These metrics are widely used in institutional benchmarking and research performance evaluation, offering a field-weighted and temporally adjusted view of influence. Only records with a minimum of one citation were included in CNCI/JNCI analysis to avoid distortion by uncited works.

### 2.4. Visualization

The specialized software VOSviewer (version 1.2011) for constructing and visualizing bibliometric networks was employed. VOSviewer enabled the development of diverse bibliometric maps, providing valuable insights into the associations among key publications, authors, and sources. Moreover, it was instrumental in generating keyword co-occurrence networks, facilitating the identification of emerging research trends and thematic patterns in the literature). Keyword normalization was performed prior to co-occurrence analysis. Variants such as “lifespan” and “life span” or “*Drosophila*” and “*Drosophila melanogaster*” were unified under a single representative term to avoid fragmentation in the network maps. This step ensured semantic consistency across the dataset.

## 3. Results

For the keywords indicated in Figure 1, 4990 articles from 2015 to 2024 were extracted without any filtering. Overall, 2203 articles met the inclusion criteria.

### 3.1. Trends in Research Volume and Impact

**Figure 2:**
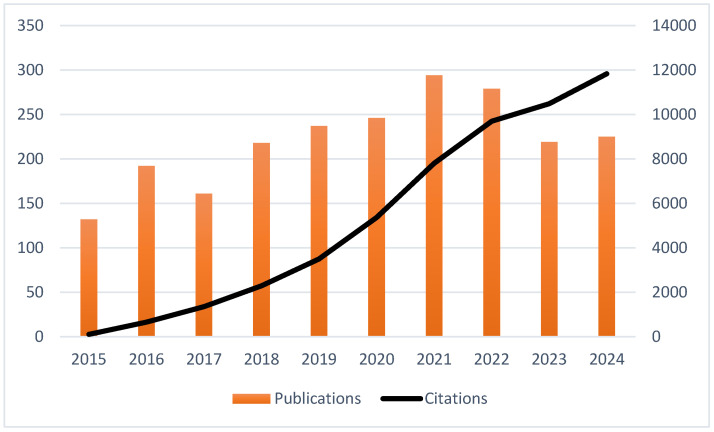
Annual trends in publication volume and citation counts on diet and longevity research (2015–2024).

The number of publications steadily increased, especially after 2017, peaking in 2021 (Figure 2). This trend reflects the increased academic interest in the field in recent years. The observed slight decline in the publication number in 2022 and 2023 may be attributed to changes in research areas, funding, or publication delays. However, over the same period, a consistent and significant increase in the number of citations was noted, reaching over 12,000 citations in 2024, indicating a growing recognition of relevant research and ongoing influence and references from earlier studies. Overall, although minor fluctuations within the publication volume were observed in recent years, the academic relevance was sustained as evidenced by the consistent increase in the number of citations.

### 3.2. Author Analysis by Number of Publications and Citations

The 10 leading authors in the topic of diet and longevity are presented in Table 1. The number of publications, total citations, h-index, citation impact, and normalized impact scores (Category Normalized Citation Impact [CNCI] and Journal Normalized Citation Impact [JNCI]) were among the several bibliometric metrics that formed the basis of the analysis. The most cited author was Rafael Carlos de Cabo (*n* = 1433), who had the highest h-index (11), indicating high productivity and substantial influence in the field. Similarly, Dudley Lamming and Sarah J. Mitchell maintained a steady presence in the field with high citation impact values (107.6 and 70.1, respectively) and robust normalized citation metrics. High visibility across disciplinary and multidisciplinary contexts can be suggested according to their strong CNCI and JNCI values. Despite having lower total citation counts, authors including Ender Buyukguzel and Andzej Bartke were also consequently listed as leading authors owing to their consistent scholarly contributions and recognition in more specialized subfields. Additional metrics for the top 20 authors, including extended citation indicators, are presented in Supplementary Table S1.

**Figure 3:**
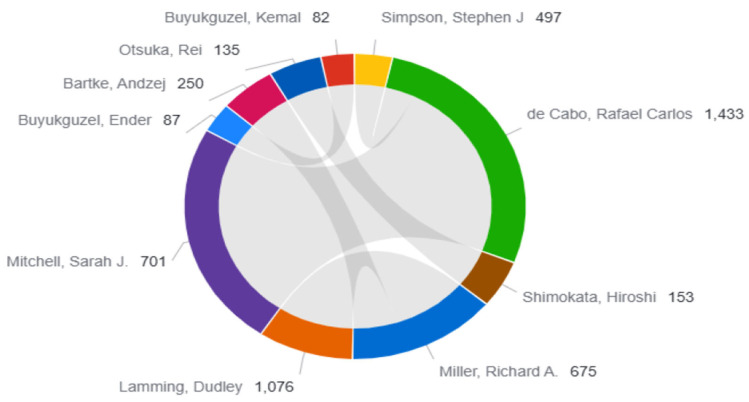
Coauthorship and citation network of the top contributing authors in diet and longevity research.

Figure 3 illustrates the coauthorship and citation network of the top contributing authors in diet and longevity research. Rafael de Cabo stands out as the most central and influential figure, with the highest citation count (*n* = 1433) and extensive collaborative ties, particularly with Richard A. Miller, Dudley Lamming, and Sarah J. Mitchell. These four authors form a densely connected cluster, reflecting not only high productivity but also a tightly integrated research network focused on mechanistic studies of caloric re-striction, mTOR signaling, and aging biology. Notably, the strong internal connectivity of this cluster suggests the presence of a core author group driving foundational advancements in the field. In contrast, contributors like Stephen J. Simpson, Ender Buyukguzel, Andrzej Bartke, and Rei Otsuka, while influential in their own domains, appear in more peripheral positions with fewer coauthorship links. This structural distinction may reflect differences in subfield specialization, institutional affiliations, or funding environments. Overall, the network visualization highlights the collaborative hierarchy within diet and longevity research, where a small number of highly cited authors play a central role in shaping scientific discourse, while others contribute more independently or within smaller clusters.

### 3.3. Country and Institution Analysis by Number of Publications and Coauthorships

As shown in Table 2, the United States (US) is the leader in the field in terms of contributions (*n* = 647) and citations (*n* = 29,507). With 481 articles, the People’s Republic of China follows the US, indicating an increased research commitment in the field. The ratio of records in the top 10 and citation impact was the highest in England, underscoring the high impact and influence of contributions originating from this country (27.74% and 101.85, accordingly). The predominance of East Asia, Western Europe, and North America may highlight the concentration of research activities in high-income and research-intensive countries with a potentially large proportion of aging population in Western Europe and North America. A detailed list of the top 20 contributing institutions, along with impact metrics and international collaboration rates, is presented in Supplementary Table S2.

**Figure 4:**
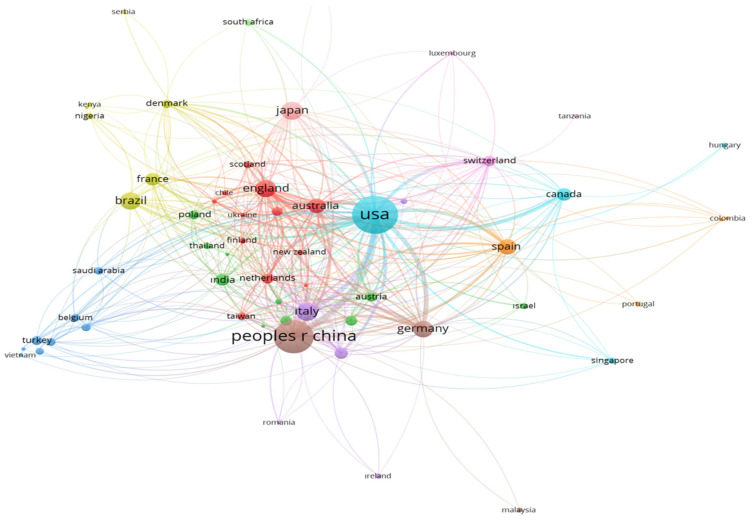
International coauthorship network: global research collaborations on diet and longevity (2015–2024).

Figure 4 displays the international coauthorship network in the field of diet and longevity research. The United States occupies the most central and dominant position in the network, reflecting its role not only as the most productive country but also as a key facilitator of global collaboration. The extensive connections radiating from the US to nearly all major nodes suggest that it serves as a hub in the knowledge dissemination process, consistent with earlier bibliometric findings in geroscience and public health. The People’s Republic of China also demonstrates a strong presence, particularly in collaboration with East Asian and European countries, highlighting its increasing investment in aging and nutrition research. The dense European cluster—including England, Germany, France, Italy, Spain, and the Netherlands—represents a regionally cohesive research network, often supported by EU-funded transnational projects that promote interdisciplinary and cross-country aging studies. Countries such as Brazil, Turkey, South Africa, and India, while located at the periphery of the network in terms of coauthorship density, nonetheless show visible collaborative links to larger nodes. This indicates an emerging integration of Global South countries into the mainstream scientific conversation on aging and diet, though their impact remains relatively modest due to systemic disparities in research funding and infrastructure. The overall structure of the network suggests a multi-polar collaboration ecosystem, where the US and Western Europe dominate core knowledge production, while countries from Asia, Latin America, and Africa are gradually increasing their participation—mirroring global demographic and epidemiologic transitions related to aging.

The leading institutions based on their research output related to the field are presented in Table 3. Among the major institutions, the University of California System was in the first place (*n* = 65), followed by Harvard University (*n* = 61) and the US Department of Agriculture (USDA) (*n* = 46). With a combined CNCI and JNCI of 299.44 (9.79–2.06), the National Institutes of Health (NIH) performed the best in terms of citation impact. Furthermore, Harvard University and NIH demonstrated the highest *h*-index. Despite a lower number of publications (*n* = 34), the University of London showed consistent output and exceptional international collaboration, with 91.18% of its publications comprising international partners.

### 3.4. Analysis of the Journals

**Figure 5:**
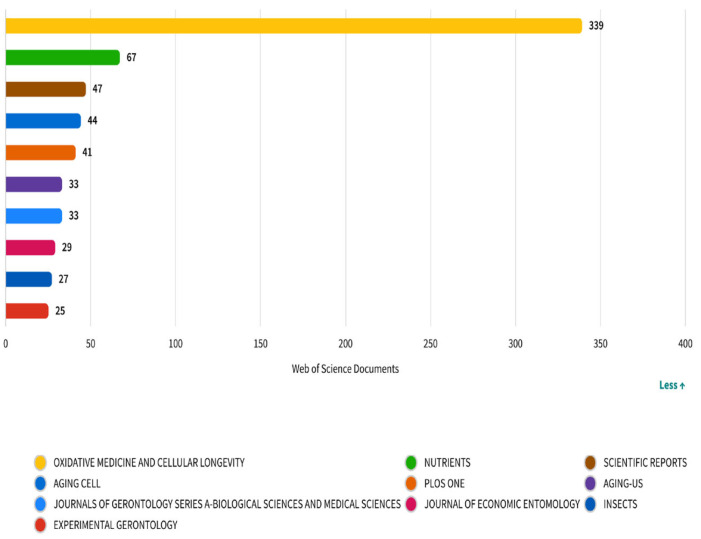
Most productive journals in diet and longevity research: number of publications per source.

Figure 5 displays the top journals publishing research on diet and longevity, based on article volume indexed in the Web of Science database. *Oxidative Medicine and Cellular Longevity* emerges as the dominant outlet with 339 publications, underscoring the field’s strong focus on oxidative stress, cellular aging, and molecular mechanisms—core topics that align with hallmarks of aging such as mitochondrial dysfunction and genomic instability. *Nutrients* (*n* = 67) and *Scientific Reports* (*n* = 47) follow, reflecting the translational and multidisciplinary expansion of the field. *Nutrients*, an open-access journal specializing in nutrition and public health, has increasingly served as a bridge between mechanistic insights and population-level dietary interventions. Meanwhile, *Scientific Reports* with its broad scope provides a platform for methodological diversity and interdisciplinary studies. Other journals such as *Aging Cell*, *Journals of Gerontology Series A*, and *Experimental Gerontology* further reinforce the prominence of geroscience within the literature. The appearance of the *Journal of Economic Entomology* and *Insects*, although more peripheral, suggests an experimental subdomain leveraging insect models like *Drosophila melanogaster* for aging-related studies. Overall, the distribution of journals illustrates the dual structure of the field: one anchored in cellular/molecular biology and the other branching into clinical nutrition and public health. This reinforces the interdisciplinary nature of diet and longevity research, encompassing both mechanistic and applied dimensions.

**Figure 6:**
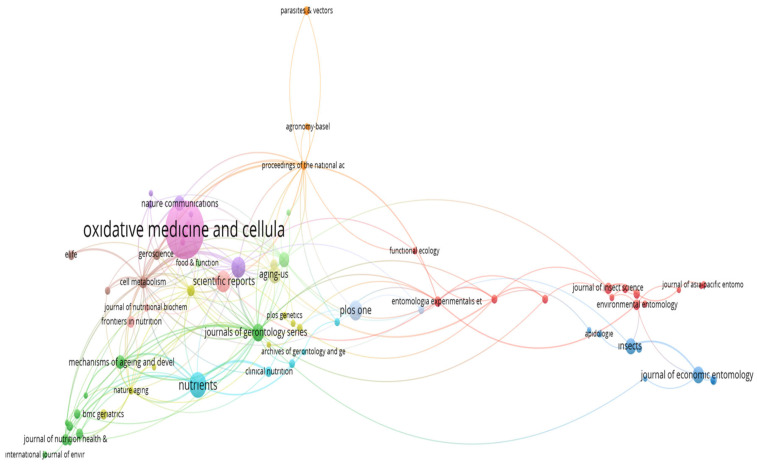
Co-citation network of scientific journals referenced in diet and longevity research.

The co-citation network of journals contributing to diet and longevity research is presented in Figure 6. At the center of the network is *Oxidative Medicine* and *Cellular Longevity*, indicating its role as a foundational source and the most frequently co-cited journal in the field. Its centrality underscores the dominance of research focused on oxidative stress, mitochondrial function, and molecular mechanisms underlying aging. Closely surrounding this core are *Nutrients*, *Scientific Reports*, *Journals of Gerontology Series*, *Aging Cell*, and *Experimental Gerontology*. The dense interconnections among these journals suggest a shared intellectual base and a cross-disciplinary dialogue between nutritional science, gerontology, and cellular biology. This reflects the convergence of experimental and clinical perspectives on longevity. Interestingly, journals such as *Insects* and the *Journal of Economic Entomology* appear in a distinct cluster on the right, indicating a niche yet coherent research area focused on model organisms like *Drosophila melanogaster*. Their close co-citation ties reflect the contribution of entomological studies to aging biology, especially in controlled dietary interventions and lifespan assays. Overall, the structure of the network highlights two dominant citation domains: (1) a central cluster driven by molecular nutrition and aging science and (2) a peripheral but cohesive subnetwork based on experimental animal models. This division reinforces the field’s interdisciplinary character and suggests that high-impact research often arises from thematic integration across molecular, translational, and ecological studies.

### 3.5. Analysis of the Keywords

**Figure 7:**
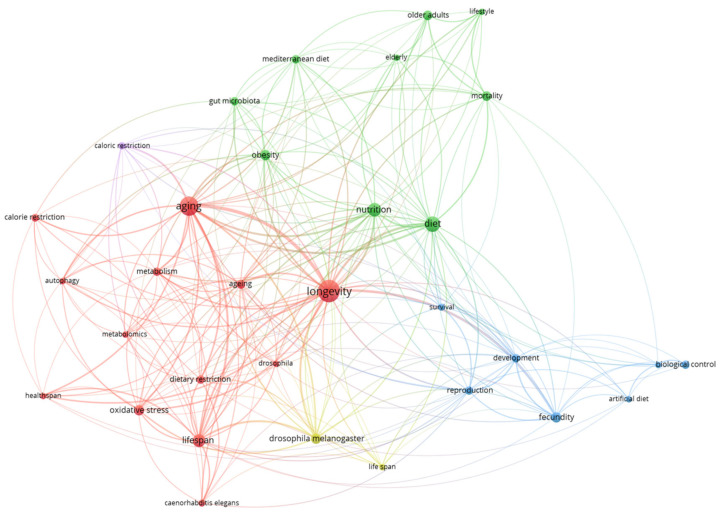
Keyword co-occurrence map: thematic clusters in diet and longevity research (2015–2024).

The keyword co-occurrence network, revealing four major conceptual clusters in diet and longevity research are illustrated in Figure 7. The red cluster, anchored by terms like aging, longevity, and lifespan, emphasizes mechanistic studies involving oxidative stress, autophagy, caloric restriction, and model organisms such as *Caenorhabditis elegans* and *Drosophila melanogaster*. The green cluster highlights population-level and public health concerns, including nutrition, obesity, mortality, and the Mediterranean diet, with growing interest in gut microbiota as a key mediator. The blue cluster focuses on reproduction and development, with terms like artificial diet and biological control, mostly from ecological and entomological research using model animals. The yellow cluster, closely related to the red, centers on *Drosophila melanogaster* and dietary restriction, reflecting genetic and nutritional intervention studies. Overall, the map demonstrates a multi-layered structure linking molecular, translational, and ecological dimensions—underscoring the field’s interdisciplinary nature and the value of integrating diverse approaches in future research. The complete keyword co-occurrence dataset, ranked by frequency and link strength, is presented in Supplementary Table S3.

## 4. Discussion

This study mainly aimed to provide a comprehensive analysis of the literature on diet and longevity from 2015 to 2024. Overall, 2203 articles were included in this study. The results showed a substantial variety of themes related to aging, a longer healthspan, and dietary trends, as well as a growing body of research interest. To the author’s knowledge, this was the first bibliometric analysis study exclusively focusing on nutrition in relation to longevity.

Interest in the subject grew over the past three decades. This growth can be explained by the increased global recognition of the significance of dietary interventions for healthy aging and an extended healthspan. A sustained interest in previous foundational works may be suggested with the steady increase in citation counts, even with a slight decline in publication output in the years that followed, which may be attributed to changing research goals, financial reallocations, or mid- and post-pandemic publishing dynamics. The increased interest, with a sharp volume in 2021, may be associated with the declaration made by the UN in May 2020, calling the upcoming decade the “Decade of Healthy Aging (2021–2030)” [12]. Following the UN Decade of Healthy Aging resolution, a global effort was developed to improve the lives of senior citizens, their families, and the communities where they reside. The interconnected areas of action encompassed changing the attitude, feeling, and behavior regarding aging; making sure that communities support the skills of senior citizens; providing person-centered integrated care and senior-responsive primary health services; and making long-term care accessible to senior citizens who need it [13]. New study designs were shaped following this declaration, placing healthy aging and longevity in the center. In 2023, despite a modest decline in the number of publications, the total citation count continued to steadily rise. With a decreased number of new articles and an increased number of citations, the trend suggests a shift from quantity to quality. This trend may present a shift in the area toward more focused studies; however, it also raises questions involving long-term research priorities and the position of previous foundational work for newer studies.

A frequently observed trend regarding the authors contributing to the area was confirmed as the productivity was not always the same as the impact. The most productive authors in terms of publication count were Amos Olalekan Abolaji (affiliated with an institution in Nigeria), followed by Simpson (affiliated with an institution in Australia), and de Cabo (affiliated with an institution in the US). In contrast, Rafael de Cabo, Dudley Lamming, and Sarah J. Mitchell were the most influential contributors based on citation impact, and all were affiliated with institutions in the US. This contrast illustrates a larger trend of scientific impact being concentrated in high-income countries with greater access to resources and networks for collaboration and visibility. Moreover, the development of significant research in this area frequently necessitates translational frameworks, experimental models, and longitudinal studies, all of which require ongoing institutional capability and resources in addition to financial support. Researchers in low- and middle-income countries may experience increased risks because of these inequalities, underscoring the significance of international research initiatives in addressing these disparities. Establishing international research consortia may make it possible to pool resources, standardize study procedures, and promote fair participation in the global agenda for longevity research. Incorporating dietary aging therapies into public health initiatives may be another option for policymakers.

The leading contribution of the United States can be attributed to its high national income and the presence of established institutions such as the University of California System, Harvard University, USDA, and NIH. The dominance of the country was not only in high productivity but also in larger citation metrics and coauthorships, emphasizing its global influence and highlighting its impact in terms of quantity and quality. The integration of the active health of the aging population as one of the leading parts of the “Healthy China” project may have supported more studies in this field, bringing China to a second place in terms of study volume [14]. Although the number of publications was not among the top rankings, the related citations were strong for studies published from the United Kingdom (UK)/England, presenting fewer studies with greater quality. The high citation metrics attributed to the UK/England could be related to the high international collaborations of the University of London as a consequence of its expanded worldwide network. Japan and Italy have shown similar numbers of studies, indicating a broadening of the research landscape and potential shifts in future leadership. The dominance of the US and the following countries in the field of studies on aging was also observed by Sabri et al. (2022) [15], Murillo et al. (2024) [16], and Wu and Zhao (2025) [17].

The highest number of articles in the field of diet and longevity was published by *Oxidative Medicine and Cellular Longevity*, followed by *Nutrients* and *Scientific Reports*, and all three were widely cited as well. According to their citation count, *Aging Cell* and *Journals of Gerontology Series A* were among the journals with the highest number of articles in the field of diet and longevity.

The adaptable structure of the area, which varied across molecular mechanisms, population health, experimental models, and model organisms, was demonstrated by the keyword co-occurrence analysis. The molecular mechanisms underlying aging and the function of diet in promoting healthy aging at the community level were the two primary axes of the complex network. Although the increasing co-occurrence of public-health-related terms indicated a recent increase in translational focus, the prevalence of experimental models shed light on the mechanistic discoveries in this discipline, which remained accepted as fundamentals.

*Drosophilia melanogaster* (*D. melanogaster*) remains one of the most investigated model organisms for studies on aging [18]. Owing to its low cost, ease of handling and maintenance, large number of offspring per adult, short life cycle and lifespan, relatively few paralogous genes, high evolutionary conservation of epigenetic mechanisms and signaling pathways, and accessibility to various tools to modify gene expression in vivo, *D. melanogaster* is a perfect model system for investigating the mechanisms of longevity and aging [19]. *Caenorhabditis elegans* (*C. elegans*), presenting a lighter presence in the domain, also remains one of the most investigated models in this field [20]. Several benefits are offered by the nematode *C. elegans*, including a short life cycle and lifespan, cost-effectiveness, high reproduction, simplicity of propagating populations of synchronized individuals, and a fully annotated genome [21]. Strong evidence from animal studies have reported that certain dietary treatments can affect aging biology and longevity, even if definitive human clinical trials of nutrition and aging are unrealistically lengthy and controlled [22]. In a comprehensive review by Park et al. (2024), dietary interventions involving particular food extracts such as pomegranates, green tea, cocoa, and berries and phytochemicals—including resveratrol, quercetin, curcumin, epigallocatechin gallate (EGCG), and anthocyanins—were summarized as extensively investigated in model organisms such as *C. elegans*, *Drosophila melanogaster*, and, to a lesser extent, mice, for their role in improving the lifespan and healthspan [23]. Supporting their broad-spectrum potential in age-related regulation, these chemicals have also been systematically linked to improvements across the acknowledged hallmarks of aging, building on data from animal models [3]. But in order to close the gap between experimental results and clinical outcomes, the further integration of rodent models, non-human primates, and well-controlled, long-term human trials are required.

The population-level health concerns of aging societies are becoming a major research priority. The focus shifts from an aging society and its changes in the age structure to a longevity society, concentrating on the advantages of longer lives through changes during aging. With the aging population, the awareness of the burden of chronic diseases and the requirement for preventive dietary strategies supporting healthy aging also increased [24]. The obesity pandemic, another significant public health problem, also provides a metabolic dysregulation similar to that observed in the normal aging process and even accelerated aging with the growing potential of obesity. Therefore, adipose tissue-targeting interventions can be valuable to better comprehending the aging process [25].

Oxidative stress is believed to be a key factor limiting the lifespan and preventing healthy aging. The damage caused by the oxidative stress to the mitochondria may be attributed to genome instability and inflammation, potentially initiating cellular senescence and cellular aging [4]. For this reason, various approaches focusing on oxidative stress are currently being investigated. The Mediterranean diet represents one of the most popular approaches to modulating oxidative stress to enhance the healthspan. Through several synergistic mechanisms, including decreased oxidative stress and chronic inflammation, enhanced endothelial and metabolic function, and the advantageous modification of the gut microbiota composition, adherence to the Mediterranean diet has been associated with longer lifespans. Its high concentration of bioactive substances, including polyphenols, unsaturated fats, and dietary fiber, together with its minimal consumption of highly processed foods and red meats are primarily responsible for these effects. The Mediterranean diet has long been considered a natural longevity model rather than a manufactured intervention, having been initially discovered in long-living individuals with exceptionally low rates of cancer and cardiovascular diseases [26].

Calorie restriction represents another growing topic of interest for researchers showing promising results for healthy aging [16]. Calorie restriction has been demonstrated to be an effective intervention to increase the lifespan and enhance the healthspan by reducing inflammation and oxidative stress through complex pathways involving changes, including the reduction in insulin levels and insulin growth factor-1 signaling, inhibition of mechanistic target of rapamycin signaling, activation of AMP-activated protein kinase and sirtuin 1, upregulation of the NAD+ pathway, and autophagy [27]. In humans and experimental models, calorie restriction and calorie restriction mimetics have been proposed as effective strategies for prolonging a healthy lifespan and slowing down aging [28]. Nonetheless, the extent, composition, and timing of nutrient intake are emerging as significant factors in maintaining human health and slowing down the aging process, in addition to calorie restriction [29,30].

Gut dysbiosis is one of the hallmarks of aging [5]. The effects of nutrition are also investigated through the perspective of host–microbiome interactions, representing a new avenue in healthy aging studies. Microbiota-targeted treatments, including fecal microbiota transplantation, have emerged as beneficial and accessible treatment alternatives for prolonging the healthspan and lifespan by working on aging-related dysbiosis and related metabolic disorders [31,32]. Gut-microbiota-targeting dietary interventions, including prebiotics, probiotics, and postbiotics, may also be useful in improving dysbiosis [33]. Reducing oxidative stress, anti-inflammaging, and anti-immunosenescence represents the main pathway for different microbiota treatments in improving dysbiosis, thereby supporting healthy aging [31].

In order to confirm results from animal models and evaluate the therapeutic significance of dietary treatments, future research should prioritize large-scale, longitudinal human investigations. Furthermore, comparative research across various ethnicities and cultural dietary customs may reveal regional approaches to healthy aging.

Diet and longevity research has gradually expanded beyond the effects of individual nutrients to encompass systemic, microbiological, and sociocultural factors that collectively influence aging trajectories. Furthermore, the inconsistent language used in the literature, such as “lifespan” versus “life span” or “*Drosophila*” versus “*Drosophila melanogaster*,” reflects a lack of consistency. This variability highlights the significance of using regulated vocabulary and may cause misunderstandings in bibliometric analysis. Furthermore, the growing body of research using experimental reproductive models presents encouraging directions for investigating the association between diet and longevity. There is a growing need for additional multidisciplinary research that combines mechanistic insights with human health outcomes to fully elucidate the underlying processes and convert the results into clinical practice.

## 5. Conclusions

This bibliometric study offers a methodical summary of how diet and longevity research has changed over the last decade. The notable increase in publication and citation trends reflect the growing interest in the mechanisms by which dietary patterns affect the biological processes of aging. Recent research has increasingly integrated molecular processes, experimental models, and population-level results, whereas earlier studies have frequently focused on particular nutrients or single pathways. Essential contributions, organizations, and publications have demonstrated that the field is becoming more complex and expanding, with particular focus on gut microbiota, oxidative stress, calorie restriction, and the Mediterranean diet. To the best of our knowledge, this is the first bibliometric study to map research trends, institutional contributions and developing conceptual clusters in a systemic manner.

Despite these advancements, the analysis shows structural imbalances in global research output and cooperation. Owing to their better financial infrastructure, institutional capability, and cooperative networks, high-income countries, including the US and the UK, continue to dominate publication and citation rankings. Low- and middle-income countries continue to publish a relatively small number of articles. The fragmented use of terminologies, including “lifespan” versus “life span”, and the inconsistent taxonomic references for model organisms constitute another challenge. Furthermore, the translational potential of the current results is hindered by the lack of longitudinal and interventional human trials, despite the abundance of mechanistic and preclinical investigations. Bridging this translational gap, to overcome the lack of this link, may be a priority in future studies. Establishing global collaboration networks, giving interventional human research first priority when it comes to financing, and promoting open-access data sharing to speed up knowledge translation are among the practical strategies to close this gap. A more egalitarian and globally relevant research agenda can be supported by employing various approaches and increasing the involvement of underrepresented regions. Another limitation of this study is that the analysis was conducted only with articles indexed in the Web of Science and published in English. The inclusion of relevant contributions from non-English sources or other databases will broaden the scope of the analysis. These discrepancies might restrict the worldwide application of findings and prevent underrepresented communities from developing nutritional aging strategies adapted to their particular regions.

This analysis lays the groundwork for future studies and multidisciplinary projects by summarizing the major contributions and changing patterns in diet and longevity studies over the last 10 years. Altogether, this study provides a solid basis for advancing multidisciplinary research and evidence-based public health initiatives, ensuring that dietary interventions are effectively included into global agendas for longevity and aging.

## 6. Limitations

This study has several limitations that should be acknowledged to contextualize its findings and guide future research. While this study thematically distinguishes between experimental (animal-based) and human population studies through keyword analysis, it does not provide a formal proportional comparison. Future bibliometric analyses may incorporate article-level classification techniques to quantify the distribution of research types across the literature. Although raw data were retrieved from proprietary databases, processed datasets used for VOSviewer visualizations and summary tables can be shared upon reasonable request to the corresponding author. Future updates of this analysis may include a supplementary Excel or CSV file listing all analyzed publications and their bibliometric attributes.

## Figures and Tables

**Table 1:** Top 10 most influential authors in diet and longevity research (2015–2024): productivity and impact metrics.

Authors	Record Count	Citation Count	H-Index	Citation Impact	CNCI	JNCI
Abolaji, Amos Olalekan	13	275	7	21.15	1.29	1.41
Simpson, Stephen J.	12	497	10	41.42	1.48	0.86
De Cabo, Rafael Carlos	11	1433	11	130.27	4.93	1.50
Shimokata, Hiroshi	10	153	6	15.3	0.97	1.25
Miller, Richard A.	10	675	10	67.5	2.39	1.19
Lamming, Dudley	10	1076	10	107.6	4.73	2.02
Mitchell, Sarah J.	10	701	8	70.1	3.00	1.19
Buyukguzel, Ender	10	87	6	8.7	0.75	1.46
Bartke, Andzej	10	250	9	25	0.86	0.71
Otsuka, Rei	9	135	5	15	1.00	1.14

CNCI, Category Normalized Citation Impact; JNCI, Journal Normalized Citation Impact.

**Table 2:** Top 10 countries/regions by research output and impact on diet and longevity (2015–2024).

Countries	Record Count	Citation Count	Records in the Top 10 (%)	Citation Impact
USA	647	29,507	150 (23.18)	45.61
Mainland China	481	17,214	74 (15.38)	35.79
United Kingdom *	158	14,313	39 (24.68)	90.59
Japan	150	10,201	17 (11.33)	68.01
Italy	149	13,507	35 (23.49)	90.65
Brazil	140	9812	14 (10.00)	70.09
England *	137	13,954	38 (27.74)	101.85
Germany	125	11,112	26 (20.80)	88.9
Spain	103	10,162	19 (18.45)	98.66
Australia	99	9982	18 (18.18)	100.83

* Due to affiliations recorded in the databases, “England” and “United Kingdom” appear as separate entries.

**Table 3:** Leading institutions in diet and longevity research: productivity, citation impact, and collaboration patterns.

Organizations	Record Count	Citation Count	H-Index	Citation Impact (CNCI–JNCI)	International Collaborations (%)
University of California System	65	10,400	23	160 (5.63–1.77)	41 (63.08)
Harvard University	61	11,428	26	187.34 (6.65–1.5)	42 (68.85)
United States Department of Agriculture	46	1186	14	25.78 (1.67–1.08)	19 (41.30)
National Institutes of Health (NIH)–USA	43	12,876	26	299.44 (9.79–2.06)	29 (67.44)
Peking University	41	1050	17	25.61 (10.91–1.29)	23 (56.10)
Chinese Academy of Sciences	41	909	15	22.17 (1.04–0.79)	18 (43.90)
University of Texas System	38	2133	18	56.13 (2.85–1.30)	15 (39.47)
Harvard T.H. Chan School of Public Health	37	10,291	20	278.14 (9.22–1.70)	26 (70.27)
State University System of Florida	35	9178	16	262.23 (7.65–2.18)	18 (51.43)
University of London	34	9504	19	279.53 (7.66–1.08)	31 (91.18)

## Data Availability

The data used in this bibliometric analysis were obtained from publicly available databases. All relevant data are included in the article.

## Supplementary Materials

The following supporting information can be downloaded at: https://www.mdpi.com/article/10.3390/nu17132119/s1. Table S1. Top 20 most influential authors in diet and longevity research (2015–2024): productivity and impact metrics. Table S2. Top 20 leading institutions in diet and longevity research: productivity, citation impact, and collaboration patterns. Table S3. Co-occurence of all keywords in diet and longevity research (2015–2024).

## Abbreviations

The following abbreviations are used in this manuscript:

AMPK	AMP-Activated Protein Kinase
CNCI	Category Normalized Citation Impact
CR	Calorie Restriction
JNC	Journal Normalized Citation
NIH	National Institutes of Health
SIRT1	Sirtuin 1
UN	United Nations
USDA	United States Department of Agriculture
VOSviewer	Visualization of the Similarities Viewer
WoS	Web of Science

## References

[1] Félix J., de Toda I.M., Díaz-Del Cerro E., González-Sánchez M., De la Fuente M. (2024). Frailty and biological age. Which best describes our aging and longevity?. Mol. Asp. Med..

[2] López-Otín C., Kroemer G. (2021). Hallmarks of health. Cell.

[3] de França N.A.G., Rolland Y., Guyonnet S., de Souto Barreto P. (2023). The role of dietary strategies in the modulation of hallmarks of aging. Ageing Res. Rev..

[4] Mensah E.O., Danyo E.K., Asase R.V. (2024). Exploring the effect of different diet types on ageing and age-related diseases. Nutrition.

[5] López-Otín C., Blasco M.A., Partridge L., Serrano M., Kroemer G. (2023). Hallmarks of aging: An expanding universe. Cell.

[6] López-Otín C., Blasco M.A., Partridge L., Serrano M., Kroemer G. (2013). The hallmarks of aging. Cell.

[7] Borras C., Ingles M., Mas-Bargues C., Dromant M., Sanz-Ros J., Román-Domínguez A., Gimeno-Mallench L., Gambini J., Viña J. (2020). Centenarians: An excellent example of resilience for successful ageing. Mech. Ageing Dev..

[8] Longo V.D., Anderson R.M. (2022). Nutrition, longevity and disease: From molecular mechanisms to interventions. Cell.

[9] United Nations (2024). World Population Prospects 2024: Summary of Results.

[10] Bautmans I., Knoop V., Thiyagarajan J.A., Maier A.B., Beard J.R., Freiberger E., Belsky D., Aubertin-Leheudre M., Mikton C., Cesari M. (2022). WHO working definition of vitality capacity for healthy longevity monitoring. Lancet Healthy Longev..

[11] Wong J.E., Fried L.P., Dzau V.J. (2023). The global roadmap for healthy longevity: United States of America national academy of medicine consensus study report, 2022. J. Econ. Ageing.

[12] World Health Organization (2020). Decade of Healthy Ageing: Baseline Report.

[13] Thiyagarajan J.A., Mikton C., Harwood R.H., Gichu M., Gaigbe-Togbe V., Jhamba T., Pokorna D., Stoevska V., Hada R., Steffan G.S. (2022). The UN Decade of healthy ageing: Strengthening measurement for monitoring health and wellbeing of older people. Age Ageing.

[14] Chen X., Giles J., Yao Y., Yip W., Meng Q., Berkman L., Chen H., Chen X., Feng J., Feng Z. (2022). The path to healthy ageing in China: A Peking University–Lancet Commission. Lancet.

[15] Sabri S.M., Annuar N., Rahman N.L.A., Musairah S.K., Mutalib H.A., Subagja I.K. (2022). Major Trends in Ageing Population Research: A Bibliometric Analysis from 2001 to 2021. Proceedings.

[16] Murillo-Cancho A.F., Lozano-Paniagua D., Manzano-Agugliaro F., Nievas-Soriano B.J. (2024). Worldwide Research on Calorie Restriction in Aging: A Bibliometric Study. Rev. Nutr. Clín. Diet. Hosp..

[17] Wu H., Zhao Z. (2025). A Bibliometric Analysis of Global Research Trends in Elderly Population Diseases. Public Health Nurs..

[18] Zakharenko L.P., Bobrovskikh M.A., Gruntenko N.E., Petrovskii D.V., Verevkin E.G., Putilov A.A. (2024). Two old wild-type strains of Drosophila melanogaster can serve as an animal model of faster and slower aging processes. Insects.

[19] Ogienko A.A., Omelina E.S., Bylino O.V., Batin M.A., Georgiev P.G., Pindyurin A.V. (2022). Drosophila as a model organism to study basic mechanisms of longevity. Int. J. Mol. Sci..

[20] Pontillo N., Lyu Y. (2025). Perception and Longevity Control in Invertebrate Model Organisms—A Mini-Review of Recent Advances. Biomolecules.

[21] Barrea L., Di Somma C., Muscogiuri G., Tarantino G., Tenore G.C., Orio F., Colao A., Savastano S. (2018). Nutrition, inflammation and liver-spleen axis. Crit. Rev. Food Sci. Nutr..

[22] Le Couteur D.G., Raubenheimer D., Solon-Biet S., de Cabo R., Simpson S.J. (2024). Does diet influence aging? Evidence from animal studies. J. Intern. Med..

[23] Park S.H., Lee D.H., Lee D.H., Jung C.H. (2024). Scientific evidence of foods that improve the lifespan and healthspan of different organisms. Nutr. Res. Rev..

[24] Scott A.J. (2021). The longevity society. Lancet Healthy Longev..

[25] Santos A.L., Sinha S. (2021). Obesity and aging: Molecular mechanisms and therapeutic approaches. Ageing Res. Rev..

[26] Dominguez L.J., Di Bella G., Veronese N., Barbagallo M. (2021). Impact of Mediterranean diet on chronic non-communicable diseases and longevity. Nutrients.

[27] Hu F.B. (2024). Diet strategies for promoting healthy aging and longevity: An epidemiological perspective. J. Intern. Med..

[28] Giacomello E., Toniolo L. (2021). The potential of calorie restriction and calorie restriction mimetics in delaying aging: Focus on experimental models. Nutrients.

[29] Austad S.N., Hoffman J.M. (2021). Beyond calorie restriction: Aging as a biological target for nutrient therapies. Curr. Opin. Biotechnol..

[30] Mihaylova M.M., Chaix A., Delibegovic M., Ramsey J.J., Bass J., Melkani G., Singh R., Chen Z., Ja W.W., Shirasu-Hiza M. (2023). When a calorie is not just a calorie: Diet quality and timing as mediators of metabolism and healthy aging. Cell Metab..

[31] Ling Z., Liu X., Cheng Y., Yan X., Wu S. (2022). Gut microbiota and aging. Crit. Rev. Food Sci. Nutr..

[32] Bárcena C., Valdés-Mas R., Mayoral P., Garabaya C., Durand S., Rodríguez F., Fernández-García M.T., Salazar N., Nogacka A.M., Garatachea N. (2019). Healthspan and lifespan extension by fecal microbiota transplantation into progeroid mice. Nat. Med..

[33] Xiao Y., Feng Y., Zhao J., Chen W., Lu W. (2025). Achieving healthy aging through gut microbiota-directed dietary intervention: Focusing on microbial biomarkers and host mechanisms. J. Adv. Res..

