# Supplementary material for: Global Trends and Developments in Diet and Longevity Research: A Bibliometric Analysis
Source: Nutrients. 2025 Jun 26;17(13):2119. doi: 10.3390/nu17132119 (PMC12251334; doi:10.3390/nu17132119)
Supplement: Supplementary file 1 [file nutrients-17-02119-s001.zip › nutrients-3698250-supplementary.pdf]

Table S1. Top 20 most influential authors in diet and longevity research (2015–2024): productivity and impact metrics

Authors	Record Count	Times Cited	H-Index	Citation Impact	CNCI	JNCI
Abolaji, Amos Olalekan	13	275	7	21.15	1.29	1.41
Simpson, Stephen J	12	497	10	41.42	1.48	0.86
De Cabo, Rafael Carlos	11	1433	11	130.27	4.93	1.50
Shimokata, Hiroshi	10	153	6	15.3	0.97	1.25
Miller, Richard A.	10	675	10	67.5	2.39	1.19
Lamming, Dudley	10	1076	10	107.6	4.73	2.02
Mitchell, Sarah J.	10	701	8	70.1	3.00	1.19
Buyukguzel, Ender	10	87	6	8.7	0.75	1.46
Bartke, Andzej	10	250	9	25	0.86	0.71
Otsuka, Rei	9	135	5	15	1.00	1.14
Buyukguzel, Kemal	9	82	6	9.11	0.81	1.59
Vina, Jose	9	137	6	15.22	0.89	0.98
Nishita, Yukiko	9	135	5	15.00	1.00	1.14
Picca, Anna	8	122	6	15.25	1.70	1.78
Raubenheimer, David	8	417	8	52.13	1.78	0.89
De Vito, Francesco	8	122	6	15.25	1.70	1.78
Calvani, Riccardo	8	122	6	15.25	1.70	1.78
Masternak, Michal M.	8	144	5	18.00	1.06	1.15
Marzetti, Emanuele	8	122	6	15.25	1.70	1.78
Papadopoulos, Nikos	8	221	6	27.63	1.32	1.13

Abbreviations: CNCI, Category Normalized Citation Impact; JNCI, Journal Normalized Citation Impact.

Table S2. Top 20 leading institutions in diet and longevity research: productivity, citation impact, and collaboration patterns

Organizations	Record Count	Times Cited	H-Index	Citation Impact (CNCI- JNCI)	International Collaborations(%)
University of California System	65	10400	23	160(5.63-1.77)	41 (63.08)
Harvard University	61	11428	26	187.34(6.65-1.5)	42 (68.85)
United States Department of Agriculture	46	1186	14	25.78(1.67-1.08)	19 (41.30)
National Institutes of Health (NIH) – USA	43	12876	26	299.44(9.79-2.06)	29 (67.44)
Peking University	41	1050	17	25.61(10.91-1.29)	23 (56.10)
Chinese Academy of Sciences	41	909	15	22.17(1.04-0.79)	18 (43.90)
University of Texas System	38	2133	18	56.13(2.85-1.30)	15 (39.47)
Hardvard T.H. Chan School of Public Health	37	10291	20	278.14(9.22-1.70)	26(70.27)
State University System of Florida	35	9178	16	262.23(7.65-2.18)	18 (51.43)
University of London	34	9504	19	279.53(7.66-1.08)	31 (91.18)
National Center for Geriatrics & Gerontology	33	409	11	12.39(0.88-0.92)	9(27.27)
CIBER - Centro de Investigacion Biomedica en Red	33	8412	17	254.91(6.97-1.21)	23(69.70)
US Department of Veterans Affairs	31	2408	21	77.68(3.32-1.38)	8(25.81)
Harvard Medical School	31	10038	17	323.81(10.55-	20(64.52)
Veterans Health Administration (VHA)	31	2408	21	1.66)	8(25.81)
Centre National de la Recherche Scientifique (CNRS)	31	749	16	77.68(3.32-1.38)	20(64.52)
Zhejiang University	30	8311	17	24.16(1.33-0.92)	15(50.00)
University of Sydney	30	8608	15	277.03(8.35-1.52)	22(73.33)
Harvard University Medical Affiliates	30	8811	17	286.93(7.81-1.24)	19(63.33)
Egyptian Knowledge Bank (EKB)	29	8161	11	293.70(9.32-1.55)	12(41.38)
				281.4(7.39-1.31)	

Table S3. Co-occurence of all keywords in diet and longevity research (2015-2024)

Rank	Keyword	Occurrences	Total link strength
1	Longevity	246	223
2	Aging	188	181
3	Diet	114	98
4	Lifespan	87	96
5	Nutrition	91	82
6	Drosophila melanogaster	57	79
7	ageing	52	53
8	Fecundity	54	51
9	Oxidative stress	41	49
10	Metabolism	35	47
11	Obesity	51	46
12	Dietary restriction	35	44
13	Reproduction	35	40
14	Development	35	38
15	Caenorhabditis elegans	28	28
16	Mediterrane diet	38	28
17	Gut microbiota	34	27
18	Mortality	47	27
19	Calorie restiriction	31	19
20	Older adults	45	18
